# Severity of aortic regurgitation assessed by area of vena contracta: a clinical two-dimensional and three-dimensional color Doppler imaging study

**DOI:** 10.1186/s12947-015-0016-5

**Published:** 2015-05-05

**Authors:** Hirotomo Sato, Tetsuro Ohta, Kimiko Hiroe, Seiji Okada, Koji Shimizu, Rinji Murakami, Kazuaki Tanabe

**Affiliations:** Department of Cardiology, Matsue City Hospital, 32-1 Nohira-cho, Matsue, Japan; Division of Cardiology, Department of Internal Medicine, Shimane University Faulty of Medicine, Izumo, Japan

**Keywords:** Doppler echocardiography, Aortic regurgitation, Vena contracta

## Abstract

**Background:**

Quantitation of aortic regurgitation (AR) using two-dimensional (2D) echocardiography, including vena contracta width (VCW) measurement, is still challenging. Three-dimensional (3D) echocardiography can directly measure the vena contracta area (VCA), regardless of the rheological characteristics. We intended to assess the possibility of 3D vena contracta area (3DVCA) as well as 2D vena contracta area (2DVCA) in the assessment of AR severity.

**Methods:**

Sixty-one patients with AR [17 female (32.7%); mean age: 74.0 ± 10.1 years] underwent 2D and 3D color Doppler echocardiography. Using conventional 2D color Doppler imaging, we measured VCW, 2DVCA, regurgitant volume (RV), and effective regurgitant orifice area (EROA). We also measured 3DVCA manually off-line from 3D full-volume color Doppler datasets for reference. Comprehensive 2D and 3D data on AR severity were successfully obtained from 52 of the 61 (85.2%) patients.

**Results:**

Significant correlations existed between 2DVCA and EROA (*r* = 0.89; p < 0.001). The cut-off 2DVCA for grading severe AR was 34 mm^2^ (area under curve: 0.95; sensitivity: 78%; specificity: 95%). Significant correlations existed between 3DVCA and EROA (*r* = 0.89; p < 0.001). The cut-off 3DVCA for grading severe AR was 32 mm^2^ (area under curve: 0.96; sensitivity: 89%; specificity: 98%). Significant correlations existed between 2DVCA and 3DVCA (*r* = 0.97; p < 0.001).

**Conclusion:**

Two-dimensional, as well as three dimensional, vena contracta area measurement is a simple technique suitable for clinical use during comprehensive Doppler echocardiographic AR assessment.

## Background

Accurate quantitation of aortic regurgitation (AR) severity is essential for effective clinical management and surgical intervention timing [1,2]. Conventional two-dimensional (2D) and color Doppler echocardiographic imaging modalities, incorporating the vena contracta width (VCW), regurgitant volume (RV), and effective regurgitant orifice area (EROA) measurement, are established methods for AR severity evaluation [3,4]. However, accurate prediction of AR severity is often challenging [5]. The vena contracta is located in the narrowest region between the proximal laminar flow acceleration zone and the distal turbulent regurgitant jet spray. Previous studies [6-8] showed VCW measurement to be a simple, reproducible method that is less dependent on loading conditions in assessing AR severity. However, the VCW measurement might over- or underestimate AR severity because the vena contracta jet shape is not always circular, but sometimes irregular or ellipsoid. Three-dimensional (3D) echocardiography can directly measure the vena contracta area (VCA): 3DVCA measurement was proven superior to VCW measurement for AR quantitation because it provides reliable assessment of the regurgitant orifice shape [9-14]. In contrast, 3DVCA processing after data acquisition is time-consuming, and the high-end echocardiographic machine capable of 3D color Doppler imaging is not always accessible. We evaluated the feasibility of measuring the cross-sectional VCA using 2D color Doppler imaging (2DVCA) in comparison with 3DVCA in assessing AR severity.

## Methods

**Figure 1:**
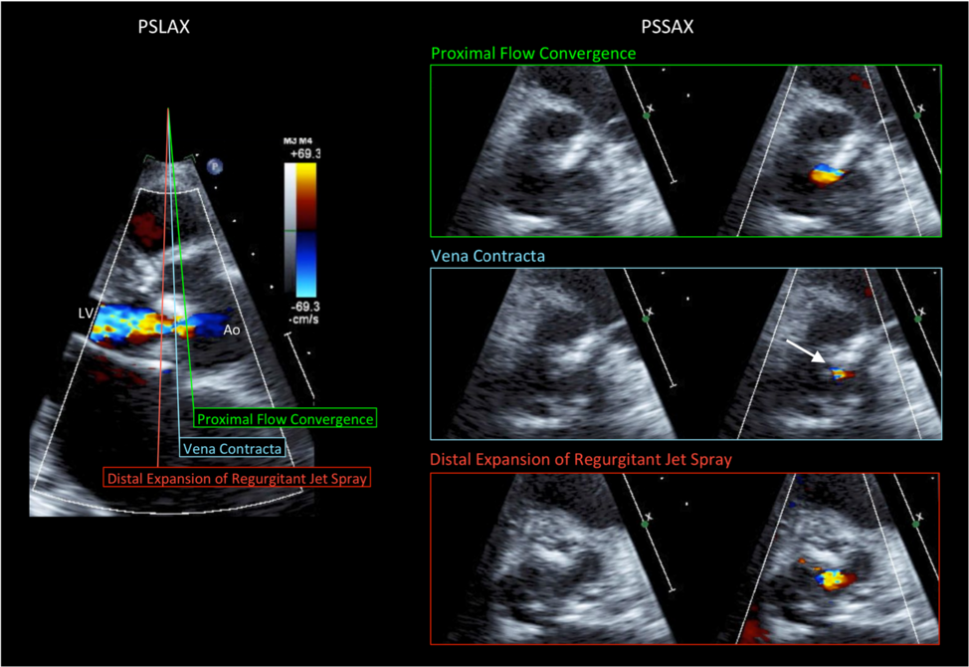
Measurement of two-dimensional vena contracta area (2DVCA) using two-dimensional color Doppler imaging. Parasternal long-axis view (left). Cross-sectional plane of the proximal flow convergence (right, top); Cross-sectional plane of the vena contracta (right, middle). The narrowest area of the jet was measured as 2DVCA (arrow). Care was taken not to include the surrounding blurred color signals as VCA by checking both color and color-suppressed images. Cross-sectional plane of the distal expansion of the regurgitant jet spray (right, bottom). PSLAX, parasternal long-axis view; PSSAX, parasternal short-axis view; Ao, aorta; LV, left ventricle.

We enrolled 61 patients referred to our institution for AR evaluation by echocardiography. Conventional echo Doppler imaging was performed using an IE-33 ultrasound machine with S5-1 and X5-1 probes (Philips Medical Systems, Andover, MA, USA). Standard measurements were performed according to American Society of Echocardiography guidelines. We measured 2DVCA, EROA (calculated by the PISA method), VCW, and RV using conventional 2D color Doppler imaging and described 2DVCA as the smallest area of the AR jet using the short-axis view during 2D color Doppler imaging while referring to a simultaneous gray scale image (Figure 1). We captured moving images at three levels (the proximal flow convergence, vena contracta, and distal expansion of the regurgitant jet spray). We measured 2DVCA from the captured images during mid diastole to minimize the effect of cardiac motion. The Nyquist limit should be maximized to distinguish the high velocity regurgitant jet from the surrounding low velocity flow, especially for eccentric flow cases [6,7]. We set the velocity range at >65 cm/sec for clear visualization of high velocity flow of vena contracta [11,12,15]. Three-dimensional echo Doppler imaging was performed using the iE-33 ultrasound machine with X3-1 and X5-1 probes, and 3DVCA measurements made manually off-line (QLAB 8.1; Philips Medical Systems). From the 3D dataset, three orthogonal planes (*x*, *y*, and *z*) were constructed. The reference lines of planes *x* and *y* were adjusted according to the largest point on the AR jet to obtain a plane exactly perpendicular to the AR jet (*z* plane). We measured the cross-sectional area at the narrowest region of the AR jet (3DVCA) in the *z* plane. Informed consent was obtained from all patients, and the protocol was approved by the hospital ethics committee.

Statistical analyses were performed using SPSS (SPSS, Inc., Chicago, IL, USA) and R (The R Foundation for Statistical Computing, Vienna, Austria). Data were expressed as mean ± standard deviation (SD), and p-values <0.05 were considered significant. To compare 2DVCA with 3DVCA and to correlate 2DVCA with EROA, we performed linear regression analyses and calculated Pearson’s correlation coefficients. To assess intraobserver variability, 2D echocardiographic data were analyzed twice off-line with a 1-week interval by the same operator. Similarly, a second observer, blinded to the results of preceding investigations, performed analyses to assess interobserver variability. Interobserver and intraobserver variability were evaluated via the intraclass correlation coefficient. Percentages of intraobserver and interobserver variability were calculated as the absolute difference divided by the average of the two measurements. Agreement was assessed using the Bland–Altman analysis. Comparison of mean values was performed using the paired *t*-test. We conducted receiver operating characteristic curve analysis to determine the optimal cut-off values in identifying severe AR (EROA: >30 mm^2^) [3-5].

## Results

Comprehensive 2D and 3D data evaluating AR severity was successfully obtained in 52 of 61 patients (85.2%). Mean patient age was 74.0 ± 10.1 years, and 17 patients were female (32.7%). Six patients (11.5%) exhibited an eccentric AR jet with bicuspid aortic valve, infective endocarditis, or aortic valve prolapse. Left ventricular diastolic diameter was 52.0 ± 8.1 mm, left ventricular end-diastolic volume was 134.7 ± 53.0 mL, left ventricular ejection fraction was 52.9 ± 10.8 % and 11 of 52 patients (21%) were severe aortic regurgitation (EROA > 30 mm^2^). Table 1 shows 2DVCA, 3DVCA, ERO, VCW, and RV.

**Table 1:** **Variables determining the severity of aortic regurgitation**

	**Mean ± SD**	**(range)**
2DVCA (mm^2^)	14.9 ± 15.6	(2–66)
VCW (mm)	3.2 ± 1.3	(1–8)
3DVCA (mm^2^)	14.3 ± 15.7	(2–72)
EROA (mm^2^)	16.9 ± 10.3	(5–46)
RV (ml)	39.8 ± 23.1	(8–104)

2DVCA, two-dimensional vena contracta area; 3DVCA, three-dimensional vena contracta area; EROA, effective regurgitant orifice area; RV, regurgitant volume; SD, standard deviation; VCW, vena contracta width.

**Figure 2:**
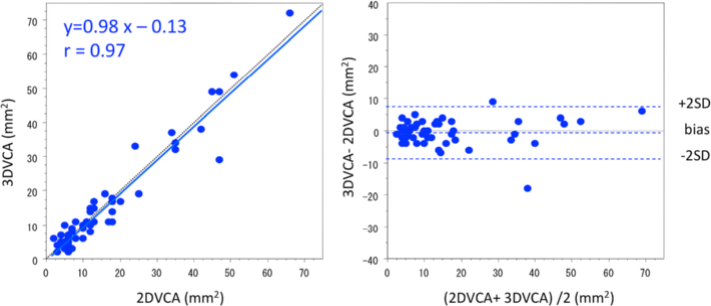
Comparison of two-dimensional vena contracta area with three-dimensional vena contracta area by correlation (left) and Bland–Altman analysis (right).

**Figure 3:**
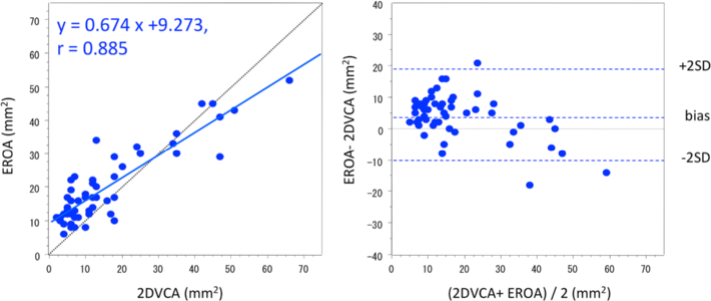
Comparison of two-dimensional vena contracta area with effective regurgitant orifice area by correlation (left) and Bland–Altman analysis (right).

Strong correlation existed between 2DVCA and 3DVCA (*r* = 0.97; p < 0.001). Bland–Altman analysis revealed an average bias of −0.36 mm^2^, and a variance (1 SD) of 4.07 mm^2^ [95% confidence interval (CI): −8.53–7.75; Figure 2]. 2DVCA also correlated with EROA (*r* = 0.89; p < 0.001). Bland–Altman analysis revealed an average bias of 4.04 mm^2^, and a variance (1 SD) of 7.12 mm^2^ (95% CI: −10.21–18.28; Figure 3). Significant correlation also existed between 2DVCA and RV (*r* = 0.80; p < 0.001) as well as 2DVCA and VCW (*r* = 0.78; p < 0.001). 3DVCA correlated with EROA (*r* = 0.89; p < 0.001). Bland–Altman analysis revealed an average bias of 4.42 mm^2^, and a variance (1 SD) of 7.57 mm^2^ (95% CI: −10.72–19.56). The optimal 2DVCA cut-off for grading AR severity was 34 mm^2^ (area under the curve: 0.95; 95% CI: 0.88-1; sensitivity: 78%; specificity: 95%) and the optimal 3DVCA cut-off for grading AR severity was 32 mm^2^ (area under the curve: 0.96; 95% CI: 0.88-1; sensitivity: 89%; specificity: 98%); for reference, EROA was >30 mm^2^ [3,4].

### **Interobserver variability**

The intraclass correlation coefficient was 0.91, and intraobserver variability was 15.9 ± 13.2%. Bland–Altman analysis revealed an average bias of 0.89 mm^2^, and a variance (1 SD) of 4.16 mm^2^ (95% CI: −7.43–9.21).

### Intraobserver variability

The intraclass correlation coefficient was 0.96, and intraobserver variability was 14.8 ± 13.5%. Bland–Altman analysis revealed an average bias of 0.98 mm^2^, and a variance (1 SD) of 3.84 mm^2^ (95% CI: −6.70–8.66).

## Discussion

We demonstrated that 2DVCA correlates with 3DVCA and EROA. Three-dimensional echocardiographic imaging facilitates direct measurement of VCA [9-14]. Using 2D echocardiography in the 2D parasternal short-axis view, VCA can be overestimated if the imaging plane is below the vena contracta (expanding portion of the jet spray), above the aortic valve (proximal flow convergence), or if perpendicular imaging plane alignment is inadequate. When measuring 2DVCA using conventional 2D color Doppler imaging, there are two major considerations. First, 2DVCA measurement must be performed at the level of the vena contracta. We primarily measured 2DVCA using the parasternal approach. We were careful to measure the cross-sectional plane of the vena contracta—the narrowest area of the jet—and not the cross-sectional planes of the proximal flow convergence or distal expansion of the regurgitant jet spray during mid-diastole (Figure 1). If the flow convergence is visible in the short-axis view, the area upstream of the vena contracta, part of the proximal isovelocity surface area, is being imaged. On the contrary, the downstream area of the AR jet is bigger than the vena contracta. For reference, we attempted to describe the complete AR jet using the long-axis view, then calculate VCA by measuring the smallest area between the proximal flow convergence and distal regurgitant jet spray in the short-axis view. It is important to begin the 2D scan at the proximal flow convergence, moving the image plane toward the regurgitant jet spray to identify the smallest area of the vena contracta.

**Figure 4:**
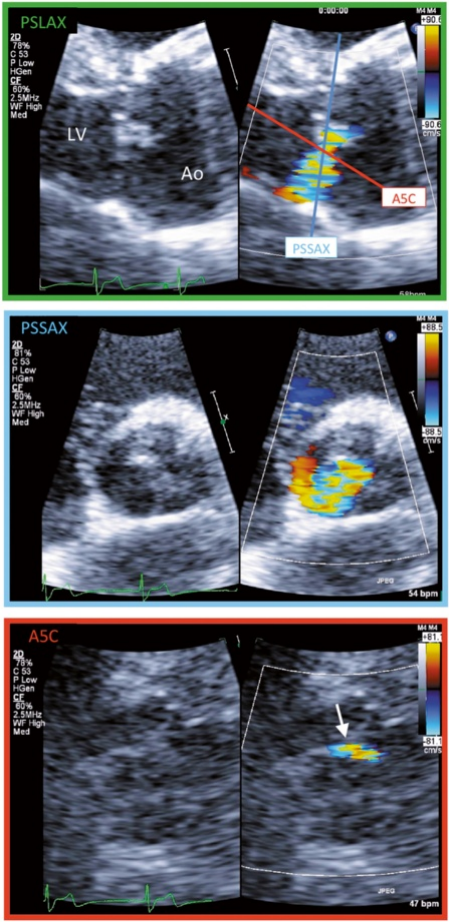
Analysis of two-dimensional color flow imaging in a patient with a bicuspid aortic valve and an eccentric aortic regurgitation (AR) jet. Parasternal long-axis view (top). After selecting the best frame for AR jet visualization, the three components of AR (the proximal flow convergence, the vena contracta, and the distal expansion of the regurgitant jet spray) were identified. Standard parasternal short-axis view (middle). This image plane was almost parallel to the AR jet, and two-dimensional vena contracta area (2DVCA) measurement was not indicated. Apical five-chamber view (bottom). This image plane was almost perpendicular to the long axis of the AR jet. The smallest area between the proximal flow convergence and distal regurgitant jet spray was measured as 2DVCA (arrow). PSLAX, parasternal long-axis view; PSSAX, parasternal short-axis view; A5C, apical five-chamber view; Ao, aorta; LV, left ventricle.

**Figure 5:**
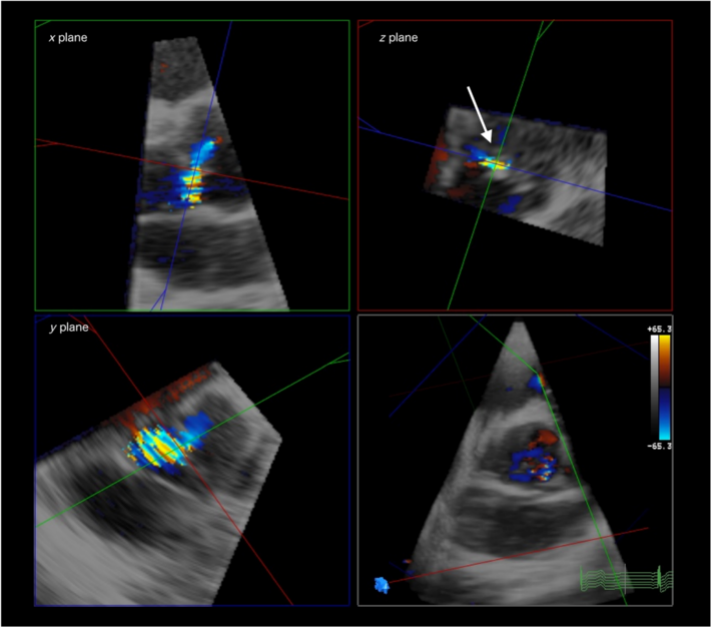
Analysis of three-dimensional color flow imaging in a patient with a bicuspid aortic valve and an eccentric aortic regurgitation (AR) jet (same patient as Figure 4). Three orthogonal planes (*x*, *y*, and *z*) were displayed using three-dimensional QLAB software. After selecting the best frame for AR jet visualization in two orthogonal long-axis views of the AR jet in the *x* and *y* planes, the dataset was cropped through the perpendicular plane of AR from the aortic side to the level of the vena contracta. Therefore, vena contracta area was visible as the narrowest region of AR in the *z* plane (arrow). Ao, aorta; LV, left ventricle.

**Figure 6:**
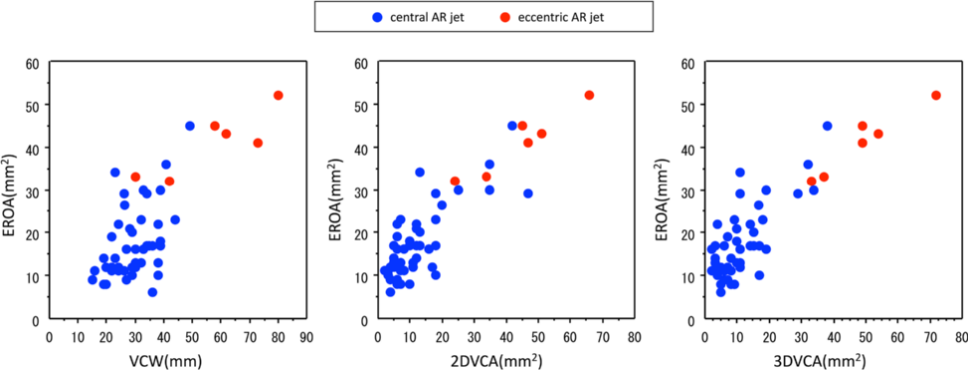
AR eccentricity affects accuracy of VCW, 2DVCA and 3DVCA. Relationship between EROA and VCW (left), 2DVCA (middle) and 3DVCA (right) for eccentric AR (red) and central AR (blue).

Second, a 2D view exactly perpendicular to the AR jet is important for measuring 2DVCA. In patients with an eccentric AR jet, we cannot accurately measure 2DVCA using the conventional parasternal short-axis view (Figure 4, top and middle). This may explain why 2DVCA is not widely accepted for accurate VCA evaluation. We attempted to project ultrasound perpendicular to the AR jet using multiple echo windows and measure 2DVCA (Figure 4, bottom) similar to 3D imaging (Figure 5). Alignment of the 2D image planes was not always perfect in older patients with reduced aortoseptal angle and in patients with eccentric AR: we measured the smallest area of 2DVCA using alternative, similar images from limited acoustic windows. In patients with calcified aortic valve lesion with eccentric AR jet, the measurement of 2DVCA may be inaccurate when valve calcification causes shadows or reverberations from parasternal window, and the measurement of 3DVCA from apical window is needed. The parasternal window provides superior axial resolution and the image from apical window is dependent on the lesser lateral resolution. In this clinical study, we used zoom mode with narrow sector angle and there was no significant difference between the measurements from either windows. The cut-off values for grading AR severity have been proposed: Fang et al. have proposed 3DVCA >60 mm^2^ to define severe AR [11]. Chin et al. reported that the cut-off value of 3DVCA was >50 mm^2^ for predicting severe AR [12]. Nozaki et al. reported that 2DERO > 30 mm^2^ corresponded to severe aortic regurgitation [15]. In patients with central AR, if a circular configuration of the VCA is assumed, then VCA was calculated by using the VCW from the following formula: VCA = π × (VCW/2)^2^. The VCW of 6 mm correspond to VCA of 28.3 mm^2^, the VCW of 7 mm correspond to VCA of 38.5 mm^2^ and the VCW of 8 mm correspond to VCA of 50.2 mm^2^. The measurement of VCA can be affected the ultrasound machine, software and machine settings. In this study, we have reported that optimal cut-off value of 3DVCA was >32 mm^2^, 2DVCA was > 34 mm^2^, for reference, ERO was >30 mm^2^. These thresholds need to be confirmed in further studies. Figure 6 shows the relationship between EROA and VCW (left), 2DVCA (middle) and 3DVCA (right). All 6 patients with eccentric AR (red) had severe AR (EROA > 30 mm^2^). VCW was >6 mm in 3 of 6 (50%) patients with eccentric AR, 2DVCA was >34 mm^2^ in 5 of 6 (83%) patients with eccentric AR and 3DVCA was >32 mm^2^ in all 6 (100%) patients with eccentric AR. The measurement of the 2D or 3DVCA could be a useful and accurate method in patients with eccentric AR.

In this study, interobserver and intraobserver variability were low and the intraclass correlation coefficient was good. To our knowledge, this is the first study to show that 2DVCA can evaluate AR severity as well as 3DVCA. Measurement of 2DVCA is a simple technique, feasible for use clinically at patients’ bedsides or in the emergency room, during comprehensive Doppler echocardiographic AR severity assessment. VCA can be measured simply by calculating the shape of regurgitant orifice as an ellipse, using the major (VCW_1_) and minor (VCW_2_) axis of the regurgitant flow, two orthogonal VCW: VCA = π × (VCW_1_) × (VCW_2_)/4 = 0.785 × (VCW_1_) × (VCW_2_), and this method may have a potential role as simple semiquantitative assessment of AR severity in patients with eccentric AR.

In this study, no patient exhibited multiple regurgitant orifices or prosthetic paravalvular regurgitation; thus, we cannot extrapolate our results to such patients. In a recent study [14], 3DVCA was reported to be a useful technique in such patients, and further studies of 2DVCA are justified. For the clinical study, we chose Doppler-derived EROA as the independent reference standard. Further studies comparing 2DVCA with magnetic resonance imaging may provide insight for assessing AR severity. The number of patients enrolled, especially those with severe AR, was small. Further studies are necessary to definitively validate this method.

## Conclusions

Significant correlation existed between 2DVCA and 3DVCA as well as between 2DVCA and ERO, RV, and VCW. Measurement of 2DVCA is a simple technique for clinical use during comprehensive Doppler echocardiographic assessment of AR severity.
